# Temperature Correction to Enhance Blood Glucose Monitoring Accuracy Using Electrical Impedance Spectroscopy

**DOI:** 10.3390/s20216231

**Published:** 2020-10-31

**Authors:** Ye Sung Lee, Minkook Son, Alexander Zhbanov, Yugyung Jung, Myoung Hoon Jung, Kunsun Eom, Sung Hyun Nam, Jongae Park, Sung Yang

**Affiliations:** 1 School of Mechanical Engineering, Gwangju Institute of Science and Technology (GIST), Gwangju 61005, Korea; lhs09444@gist.ac.kr (Y.S.L.); azhbanov@gist.ac.kr (A.Z.); 2 Department of Biomedical Science and Engineering, Gwangju Institute of Science and Technology (GIST), Gwangju 61005, Korea; son41976392@gist.ac.kr (M.S.); jyg4789@gist.ac.kr (Y.J.); 3 Healthcare Sensor Lab, Device Research Center, Samsung Advanced Institute of Technology, Samsung Electronics Co. Ltd., Gyeonggi-do 16678, Korea; mh5.jung@samsung.com (M.H.J.); k.eom@samsung.com (K.E.); sh303.nam@samsung.com (S.H.N.); jongae.park@samsung.com (J.P.)

**Keywords:** blood impedance, electrochemical impedance spectroscopy, electrical properties, blood glucose measurement, temperature correction

## Abstract

Electrical methods are among the primarily studied non-invasive glucose measurement techniques; however, various factors affect the accuracy of the sensors used. Of these, the temperature is a critical factor; hence, the effects of temperature on the electrical properties of blood components are investigated in this study. Furthermore, the changes in the electrical properties of blood according to the glucose level are corrected by considering the effects of temperature on the electrical properties. An impedance sensor is developed and used to measure whole blood impedance in 10 healthy participants at various temperatures and glucose levels. Subsequently, the conductivities of the plasma and cytoplasm were extracted. Changes in the electrical properties of the blood components are then analyzed using linear regression and repeated measures ANOVA. The electrical conductivities of plasma and cytoplasm increased with increasing temperatures (plasma: 0.0397 (slope), 0.7814 (*R^2^*), cytoplasm: 0.014 (slope), 0.694 (*R^2^*)). At three values of increasing glucose levels (85.4, 158.1, and 271.8 mg/dL), the electrical conductivities of the plasma and cytoplasm decreased. These tendencies are more significant upon temperature corrections (*p*-values; plasma: 0.001, 0.001, cytoplasm: 0.003, 0.002). The relationships between temperature and electrical conductivity changes can thus be used for temperature corrections in blood glucose measurement.

## 1. Introduction

Diabetes is a chronic disease with a globally increasing prevalence, and diabetic patients need to perform self-management, such as diet control, exercise, and weight maintenance during treatment [1,2]. To help manage diabetes, the blood glucose levels are periodically monitored via a glucometer [3,4]. Currently, the most widely used method for measuring blood glucose levels is the finger stick. This method is minimally invasive and measures glucose levels by collecting blood from the capillaries at the fingertips. Although the importance of continuous blood glucose measurement has often been emphasized clinically, the conventional method is difficult to use for repeated measurements owing to the pain experienced during blood collection. Therefore, studies on non-invasive blood glucose measurements have been actively performed [5].

Optical and electrical methods have been mainly studied as non-invasive blood glucose measurement methods [6]. For each method, there have been attempts at commercialization. Although the electrical method is more suitable than the optical method for developing portable equipment, to the best of our knowledge, these techniques have not been successfully commercialized because both methods are less accurate than the finger-stick method [7]. The decreased measurement accuracy is attributable to various factors. To improve the accuracy of measuring the blood glucose level, the effects of these factors should be individually investigated and controlled or corrected [8,9]. These factors can be broadly classified into extrinsic and intrinsic factors. The extrinsic factors mainly include skin conditions [10] and posture [11], and the intrinsic factors include blood temperature [12,13,14,15,16], blood flow rate [17,18,19,20], and hematocrit [13,14,21].

Numerous studies have investigated the effects of the intrinsic factors using in-vitro experiments. In particular, temperature causes a direct change in the electrical properties of blood. Some of the studies quantifying this effect are as follows. Mohapatra et al. [13] confirmed that the resistivity of blood decreased with the temperature at 100 kHz. Although experiments were performed using pig blood, Tjin et al. [14] reported that resistivity decreased by 22% as the temperature increased from 33 °C to 42 °C. Jaspard and Nadi [12] reported that the relative permittivity and conductivity of whole blood changed by 0.3%/°C and 1%/°C, respectively, on average in the measured frequency band (1 MHz–1 GHz). Ley et al. observed changes in the dielectric properties of whole blood according to the temperature (30–50 °C) in the high-frequency band (0.5–7 GHz). The reported electrical conductivity changes with temperature were small, and those for permittivity were large in contrast to the low-frequency range [15]. Furthermore, some studies observed changes in the electrical properties of plasma, a component of blood, with temperature. Visser et al. [20] showed that plasma conductivity at 100 kHz demonstrated a quadratic relationship with temperature, and the plasma conductivity increased with temperature. These studies could be used to predict the electrical properties of in-vivo blood in the body temperature range.

Voltages in the frequency range from kilohertz to megahertz are mainly used to measure the impedance and electrical properties of whole blood [22,23,24,25]. Because whole blood is a cell-suspended heterogeneous mixture, its impedance is not purely resistive but dielectric in nature. Therefore, the arrangement of the electric flux varies according to the frequency of the applied alternating current (AC) voltage, and the whole blood impedance is frequency dependent. Numerous studies have been conducted to investigate impedance changes using specific frequencies according to various factors and glucose levels; however, to the best of our knowledge, these impedance changes are quantified differently for each frequency owing to the frequency-dependent characteristics of the impedance of whole blood [26,27]. Accordingly, it is necessary to perform experiments at each frequency to utilize the results for temperature correction in the electrical method for glucose monitoring.

Conversely, there are frequency-independent values among the electrical characteristics of blood. Whole blood can be mainly classified into plasma, erythrocyte cytoplasm, and erythrocyte membrane. Unlike the dielectric properties of whole blood, each of these elements is not a heterogeneous mixture. Thus, it can be assumed that the dielectric properties of the individual elements are constant in the frequency range of 1 GHz or less [22,23,24,25], and this assumption is often used in practical applications [24]. Therefore, we extracted the frequency-independent values from the blood impedance and quantified the effects of temperature on the electrical properties of blood.

The electrical properties of the plasma and the other elements were extracted and quantified using whole blood samples collected from healthy people to investigate the electrical changes in the components of blood according to temperature. During the experiments, the other intrinsic factors were controlled using fixed values of blood flow rate and hematocrit. Furthermore, these data were used to correct the temperature factor in blood glucose level experiments and to investigate the effects of the blood glucose levels on the electrical properties of blood.

## 2. Materials and Methods

### 2.1. Impedance Sensor

**Figure 1:**
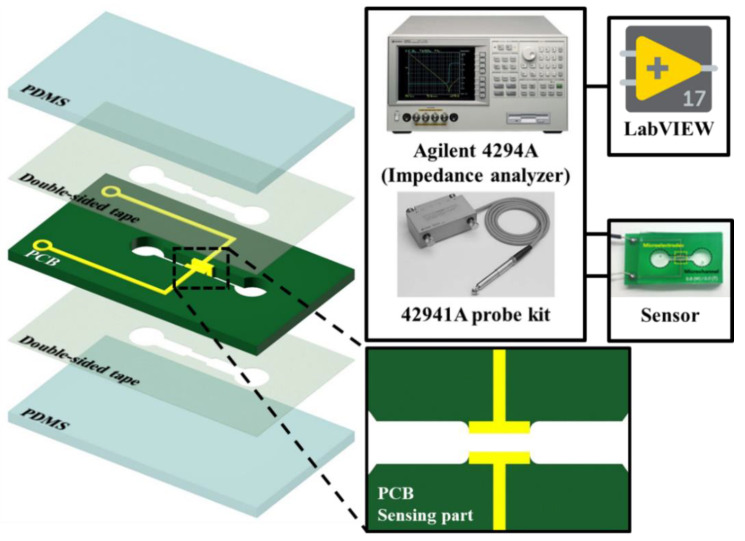
Schematic view of the proposed system. In the case of the temperature-dependence test, an impedance analyzer equipped with an impedance probe kit is connected to the electrode part of the sensor. The impedance is obtained by sweeping the AC voltage frequency (0.5 V, 1 kHz to 110 MHz) of the connected impedance analyzer while the sample flows in the channel. The entire system is controlled using software based on the LabVIEW program.

The impedance measurement sensor used in this study consisted of a channel-shaped printed circuit board (PCB) and polydimethylsiloxane (PDMS; Sylgard 184 A/B, Dow Corning, Seoul, South Korea) covers on the top and bottom (Figure 1). In this PCB, a pair of electrodes were located on the side surface in the middle of the channel. A double-sided tape manufactured in the shape of a channel using a cutting plotter (CE6000-40, Graphtec, Tokyo, Japan) was used to bond the PCB to the PDMS covers. The PDMS covers were then bonded to the double-sided tape using oxygen plasma treatment (COVANCE, Femto Science, Gyeonggi, South Korea). The PCB was manufactured in a channel shape, and two holes were made in the upper PDMS cover to create an inlet and an outlet for the sample fluid.

### 2.2. Sample Preparation

The protocol of this study was approved by the Institutional Review Board of Gwangju Institute of Science and Technology (20190510-BR-45-07-02). Ten healthy subjects were recruited, and experiments were performed using blood collected from these subjects. All subjects provided informed consent. After collection, the blood was maintained in an ethylenediaminetetraacetic acid (EDTA) tube, stored at 4 °C for 2 h, and then used in the experiment. For the glucose level tests, the blood was dispensed into three tubes, and two of the tubes were injected with a 50% dextrose solution (Daihan Pharm Co., Seoul, Korea) to adjust the blood glucose concentration, increasing it by 100 mg/dL and 200 mg/dL as compared to the original blood glucose concentration.

### 2.3. Experimental Setup

To extract the electrical characteristics of whole blood, an impedance probe kit (42941A, Agilent, Santa Clara, CA, USA) or an impedance analyzer fixture (16047E, Agilent, Santa Clara, CA, USA) equipped with an impedance analyzer (4294A, Agilent, Santa Clara, CA, USA) was connected to the electrode part of the sensor. Using a syringe pump (neMESYS, CETONI GmbH, Korbußen, Germany), whole blood was flowed into the channel at a constant flow rate. The impedance was obtained by sweeping the AC voltage frequency (0.5 V, 1 kHz to 110 MHz) of the connected impedance analyzer while flowing the sample. For temperature control, a forced convection oven (thermal chamber, OF-12GW, JEIO TECH, Daejeon, Korea) was used, and the temperature was measured in real time during impedance measurements via an inserted thermocouple (type-T, copper-constantan) in the outlet of the sensor. In addition, the entire system was controlled using software based on the LabVIEW program (National Instruments, Austin, TX, USA).

### 2.4. Extraction of Dielectric Properties

**Figure 2:**
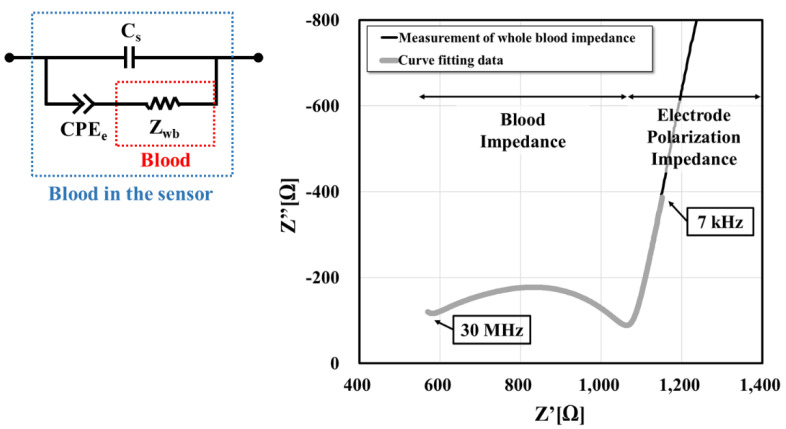
Curve fitting data with an electrical equivalent circuit. Curve fitting was performed using an equivalent circuit to extract the electrical properties of the blood from the measured impedances. For the impedance fitting, the 7 kHz to 30 MHz range of the impedance graph was used in the temperature dependence test.

The measured data from the impedance analyzer included not only the impedance of a sample (blood), but also the influence of the sensor’s geometrical shape, the parasitic impedance of the sensor itself, and electrode polarization effect. Thus, the cell constant (α) was used to consider the effect of the sensor’s geometry [25]. The stray capacitance (C_s_) was used to consider the sensor’s parasitic impedance. The effect of electrode polarization arises at the interface between the liquid and the electrode surfaces, which is common in two-electrode measuring cells. This phenomenon can be taken into account by introducing a constant phase element (CPE_e_); e is the notation for the electrode. The values of α and Cs were extracted from the impedance of air and de-ionized (DI) water (0.5 V, 40 Hz to 110 MHz). Then, as shown in Figure 2, curve fitting was performed using an equivalent circuit from our previous study to extract the electrical properties of the blood from the measured impedance (0.5 V, 1 kHz to 110 MHz) [25]. In that study, we found that the best equivalent circuit model was basically composed of C_s_, CPE_e_, and Z_wb_ (intrinsic blood impedance). The lumped parameters contributing to Z were determined numerically and compared with the experimental data, resulting that the six elements (C_e_, R_e_, C_p_, R_p_, R_i_, CPE_i_) model was the best fit with the experimental data. Thus, we have borrowed and used the six elements model (Figure 6D), [25]) and denoted them as a whole by Z_wb_ in current study.

**Figure 3:**
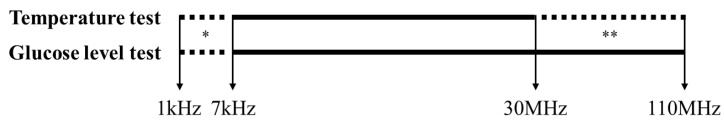
The frequency range for the fitting of each test. * This range is not used in both experiments because of an unknown noise in the frequency range of the 1 kHz to 7 KHz. ** This range is not used in temperature test because a probe kit generates noise in the frequency range of the 30 MHz to 110 MHz.

The frequency range for the fitting of each test is shown in Figure 3. The impedance in the range of 7 kHz–30 MHz is used for fitting in the temperature test. The impedance in the range of 7 kHz–110 MHz is used for fitting in the glucose level test.

The permittivity and conductivity of plasma, membrane, and cytoplasm were used for nonlinear least-squares fitting between the measured and calculated blood impedance curves. The utilized parameters are the previously extracted α, C_s_, and CPE_e_, which are used as fixed constants, as well as the electrical conductivity and dielectric constant of the plasma, cytoplasm, and membrane, which are variables for which we applied literature values as the initial values. In addition, the measured hematocrit of the whole blood sample; the x, y, and z size of the erythrocytes known in the literature; and the thickness of the erythrocyte membrane were used as fixed constants [23,24,25,28]. The fitting procedure was detailed previously [22,25].

### 2.5. Temperature and Glucose Level Tests

To observe the changes in dielectric properties with temperature, experiments were conducted using a thermal chamber. The syringe pump, impedance probe, and sensor were placed inside the thermal chamber, and the impedance change was measured while increasing the temperature. The impedance was measured every 0.5 to 1 °C step of the sample temperature measured in real time using a thermocouple from 20 °C to 40 °C. The flow rate was maintained at 3 mL/h, and the hematocrit was measured for each sample with a microcentrifuge (MF300, Hanil Science Medical, Gimpo-si, South Korea).

To investigate the changes in the dielectric properties with glucose levels, the electrode part of the sensor was connected to an impedance analyzer fixture. The experiment was performed at room temperature (about 22 °C) without temperature control, and the other experimental conditions were maintained the same as those of the temperature experiment. The prepared sample was sequentially injected from a low concentration to a high concentration with a syringe pump, and the changes in the blood impedance according to the blood glucose levels could thus be measured.

### 2.6. Statistical Analysis

Linear regression analysis was performed, and *R*^2^ was calculated for the changes in the electrical properties according to the blood temperature for all subjects and separately for each subject. A correlation analysis was performed on the changes in the dielectric constant of the plasma and cytoplasm with temperature, and the *R*-value between the temperature and dielectric constant was calculated. Repeated measures analysis of variance (ANOVA) was performed because the changes in the electrical properties of the blood according to glucose levels were measured repeatedly after varying only the blood glucose for the same blood sample. A *p*-value of < 0.05 was considered to be statistically significant.

## 3. Results

### 3.1. Temperature Dependence Test for the Electrical Properties of Whole Blood

**Figure 4:**
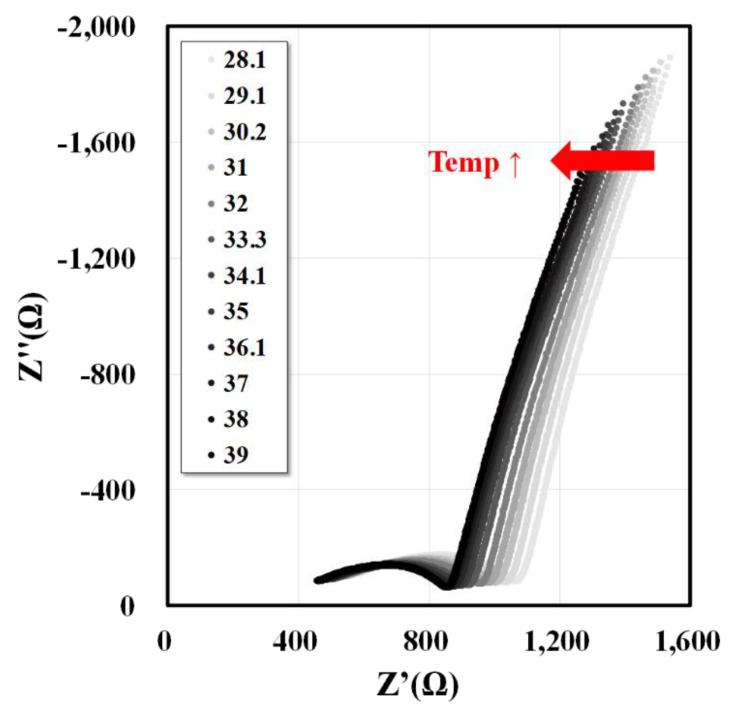
Whole blood impedance changes with increasing temperature (frequency range of 1 kHz to 30 MHz). The whole blood impedances were obtained at each temperature and graphed as Nyquist plots. The graphs show a tendency to move in the negative x-axis direction (arrow direction) as the temperature increases. The x-axis represents Z’ (resistance) of the impedance, and the y-axis represents Z’’ (reactance) of the impedance.

**Figure 5:**
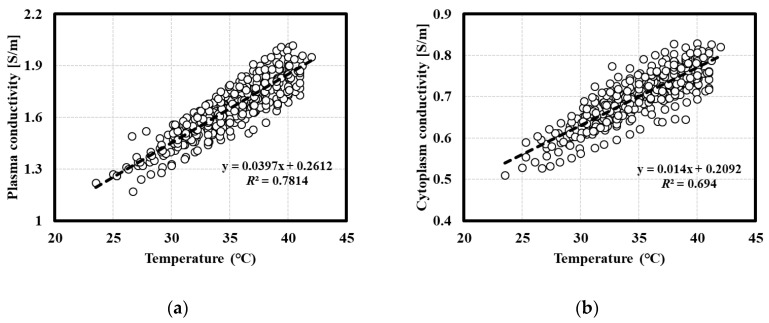
Temperature dependence of the conductivities of the plasma and cytoplasm. The electrical conductivities of the plasma and cytoplasm were extracted for 10 subjects. (**a**) Plasma conductivity. Linear regression was performed, and *R*^2^ was calculated $\left(0.0397T+0.2612;\;R^{2}=0.7814\right)$; (**b**) Cytoplasm conductivity. Linear regression was performed, and *R*^2^ was calculated ($0.014T+0.2092;\;R^{2}=0.694$).

Changes in the electrical conductivities of the plasma and cytoplasm were observed and quantified along with the temperature increase. The whole blood impedances were obtained at each temperature and graphed as a Nyquist plot. The curve shows a tendency to move in the negative x-axis direction (arrow direction) as the temperature increases (Figure 4). The x-axis represents Z’ (resistance) of the impedance, and the y-axis represents Z’’ (reactance) of the impedance. Here, Z’ decreases with increasing temperature; thus, the resistance component of the impedance decreases. The electrical conductivities of the plasma and cytoplasm were extracted for 10 subjects using this method. The electrical conductivities of the plasma and cytoplasm increased with temperature. Subsequently, the electrical conductivity data were linearly fitted to the temperature. The electrical conductivity increased with increasing temperature, and the linear relationship for the plasma and cytoplasm conductivities in all data of ten subjects are given as $0.0397T+0.2612\;\left(R^{2}=0.7814\right)$ and $0.014T+0.2092\;\left(R^{2}=0.694\right)$, respectively (Figure 5). In addition, the relationship between the temperature and conductivity used for the temperature correction for each subject was extracted (Table S1).

### 3.2. Glucose Level Dependence Test for the Electrical Properties of Whole Blood with Temperature Correction

We observed the changes in the electrical conductivities of the plasma and cytoplasm according to changes in the blood glucose levels without the thermal chamber and evaluated the correction effects of temperature based on the previous experiment.

Blood samples were obtained from 10 subjects, and each sample was prepared with three glucose levels. The average values of the glucose levels from whole ten subjects were as follows: 85.4 ± 9.1 mg/dL (Level 1), 158.1 ± 9.7 mg/dL (Level 2), and 271.8 ± 14.3 mg/dL (Level 3). From these, the electrical conductivities of the plasma and cytoplasm for each subject were extracted, as shown in Figure S1(a) gray blocks and Figure S1(b) gray blocks, respectively. In most samples, the electrical conductivities of the plasma and cytoplasm decrease as the glucose levels increase. The data with the temperature corrected to 22 °C are shown in Figure S1a red blocks and Figure S1b red blocks. The decreasing conductivities with increasing glucose levels became more obvious by the calibration process.

**Figure 6:**
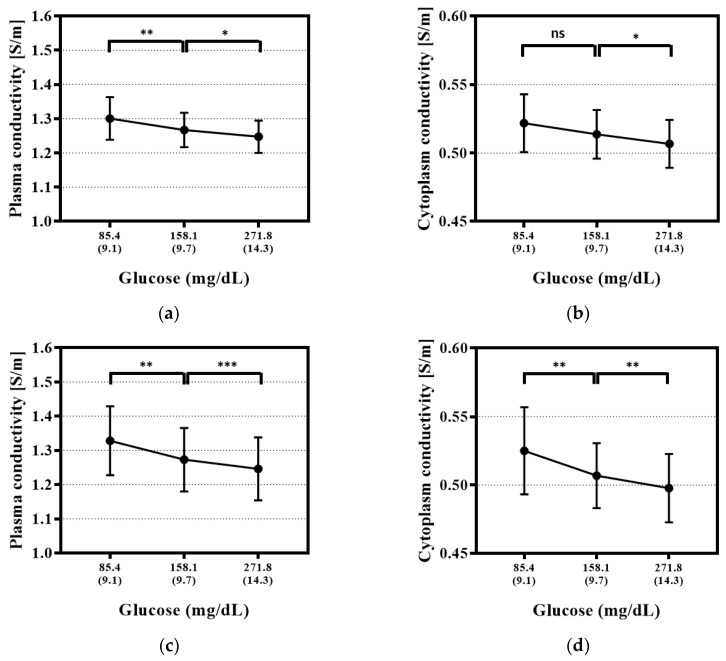
Conductivity trends for the plasma and cytoplasm according to the glucose levels. According to the glucose levels, the conductivity trends according to the glucose levels were integrated from the 10 subjects and analyzed. Each number of asterisks corresponds to the following *p*-values: * *p*-value < 0.05, ** *p*-value < 0.01, *** *p*-value < 0.001. ns: not significant. (**a**) Plasma conductivity trends before temperature correction. (**b**) Cytoplasm conductivity trends before temperature correction. (**c**) Plasma conductivity trends after temperature correction. (**d**) Cytoplasm conductivity trends after temperature correction.

According to the glucose levels, the conductivity trends were integrated from the whole ten subjects and analyzed (Figure 6). With or without temperature correction, the plasma conductivity decreased significantly with increasing blood glucose levels in all data of ten subjects. However, temperature correction renders these trends more remarkable (improvement in the *p*-value of Level 1 to 2, Level 2 to 3: 0.008 to 0.001, 0.016 to 0.001, Figure 6a,c). In the cytoplasm, the conductivity decreases as the blood glucose level increases, and the effect of temperature correction is especially effective for the Level 1 to 2 section (improvement in *p*-values of Level 1 to 2, Level 2 to 3: 0.075 to 0.003, 0.009 to 0.002, Figure 6b,d).

## 4. Discussion

The experimental results show that increasing temperature decreases the whole blood impedance. Because the resistance is inversely related to electrical conductivity, the electrical conductivity of whole blood increases with increasing temperature. Whole blood is a heterogeneous mixture; in this type of mixture, there are mainly extracellular currents at low frequencies (Hz–kHz), and the ratio of intracellular currents increase with increasing frequency (GHz band). Thus, the impedance and dielectric properties of blood have frequency-dependent values. In addition, when analyzing the changes according to the temperatures for whole blood, such changes also vary depending on the frequency [12]. Therefore, we investigated the changes in the conductivities of plasma and cytoplasm with temperature. These can be assumed to be frequency-independent for frequency bands below 1 GHz [22,23,24,25]. As shown in Figure 5, the electrical conductivities of the plasma extracted from the whole blood impedance and that of the cytoplasm increase with temperature. These phenomena are expected to have occurred in the following sequence of steps. Increasing temperature decreases the viscosity (*η*) of the fluids, such as plasma and cytoplasm. Thus, the mobilities of the ions in the fluid increase, yielding an increase in the electrical conductivity [27]. The electrical conductivities of aqueous solutions increase experimentally by 1–3% for every 1 °C with increasing temperature [29]. This relationship can be expressed as a linear equation, as in previous studies. In addition, if the relationships among the electrical conductivity (σ), diffusion coefficient (*D*), and viscosity (*η*) are utilized, the relationship between conductivity and temperature can also be understood mathematically as follows [30].

First, the Nernst–Einstein equation shows the proportionality between σ and *D* in an aqueous solution:

(1) $$\sigma =\left(\frac{F^{2}z^{2}c}{R}\right)\frac{D}{T}=\left(\mathrm{constant}\right)\frac{D}{T}\;,$$

where *F* is the Faraday constant, z is the electrical charge, c is the molar concentration of the dissolved ion(s), *R* is the gas constant, and *T* is the temperature in Kelvin.

Second, for low Reynolds numbers (Re = 0.28 in this experiment), the relationship between *D* and *η* in the aqueous solution can be obtained by the Stokes–Einstein equation:

(2) $$D=\frac{k_{B}T}{6\pi \eta r}\;⟹\;\eta =\frac{k_{B}}{6\pi r}\frac{T}{D}=\left(\mathrm{constant}\right)\frac{T}{D}\;,$$

where *k*_B_ is the Boltzmann constant, and *r* is the hydraulic radius of the diffusing ion.

Combining these two relationships, the following expression is obtained:

(3) $$\frac{\sigma_{1}}{\sigma_{2}}=\frac{D_{1}/T_{1}}{D_{2}/T_{2}}=\frac{\eta_{2}}{\eta_{1}}\;.$$

Here, 1 and 2 are notations to indicate two different temperatures.

Several empirical equations have been published for the relationship between viscosity and temperature of a fluid, which shows that the temperature is inversely proportional to viscosity for most fluids [31,32,33,34]. The increase in temperature reduces the fluid cohesive force and therefore the viscosity [35]. Accordingly, increasing the temperature decreases the fluid viscosity and consequently increases the electrical conductivity of the fluid. The relationship between conductivity and temperature can be expressed in various forms; however, most relations are represented by empirical formulas [36]. This relation is expressed as a linear equation in this study, as in conventional methods, and it can be used for aqueous solutions in the given temperature range (20–40 °C) [37].

In addition, for the permittivities of the cytoplasm and membrane, the trends are difficult to specify as a result of the correlation analysis with temperature. The results of the linear regression analysis show that the *R*^2^ values are small, i.e., 0.02 and 0.05, respectively (Figure S2). Thus, as in the previous studies, the permittivity does not show any significant tendency with temperature compared to conductivity [12].

Next, as the blood glucose levels increase, the conductivities of the plasma and cytoplasm decrease (Figure 6). These phenomena are expected to occur as follows. The glucose molecule is not an ion, and the viscosity of blood would increase with increasing concentrations of glucose [38,39]. In addition, glucose is dissolved in the plasma initially and then moves into the erythrocytes by facilitated diffusion. Then, the viscosity could increase in both the plasma and cytoplasm. Therefore, electrical conductivity could decrease as ion mobility decreases. Although the rate of change of electrical conductivity according to the glucose level is different for each subject (Figure S1), from the integrated data, the electrical conductivity is observed to decrease with temperature (plasma: *p*-values of Level 1 to 2, Level 2 to 3: 0.008, 0.016; cytoplasm: *p*-values of Level 1 to 2, Level 2 to 3: 0.075 (not significant), 0.009). The permittivity was also extracted to observe changes according to glucose levels; however, the changes were not significant (Figure S3).

The changes in electrical conductivity according to individual blood glucose levels become more obvious through temperature correction (Figure 6 (c), (d)). In the integrated data, the electrical conductivity is observed to significantly decrease according to the glucose levels. The glucose level experiments are performed at room temperature, and the range of temperature changes is insignificant at approximately ±1 °C. The results after temperature correction show a more significant decrease (plasma: *p*-values of Level 1 to 2, Level 2 to 3: 0.001, 0.001; cytoplasm: *p*-values of Level 1 to 2, Level 2 to 3: 0.003, 0.002); therefore, the process for correcting or controlling the effects of temperature may be required when measuring blood glucose levels.

There is a need for non-invasive measurement of blood glucose levels; however, when the sensor is located outside the body, accuracy, repeatability, and stability of blood glucose level measurements cannot be guaranteed owing to various extrinsic and intrinsic factors. Thus, to quantify the changes due to temperature, correction or control is applied by analyzing the effects of the intrinsic factors in-vitro in whole blood, such as the blood flow rate and hematocrit. In addition, if in-vivo experiments on the extrinsic factors, such as skin characteristics, are performed based on the previous correction results, the proposed blood glucose level measurement technique is expected to be clinically applicable.

There are some limitations in this study. First, because it is performed in-vitro, the hormonal effects and physiological responses of the subjects are not considered. Second, the values of the electrical conductivities from the plasma and cytoplasm are calculated from the impedance and not measured directly. Moreover, in these calculations, numerical modeling for the plasma and cytoplasm is already assumed, so the extracted conductivity values may vary according to the modeling method chosen.

## 5. Conclusions

A basic study on the non-invasive measurement of blood glucose levels was conducted. The changes in the blood impedance and electrical conductivity were analyzed according to the extrinsic factors, and these extrinsic factors were corrected by analyzing the changes in the glucose levels. A linear relationship was derived between the plasma conductivity and cytoplasm conductivity with increasing temperature. Next, the electrical conductivities of each of the components of blood were extracted from changes in whole blood impedance according to the glucose levels. By integrating the obtained results into the glucose level experiments, the conductivity trends were observed to be more significant when the influence of one of the extrinsic factors, namely temperature, was corrected. The changes in the permittivities of each of the components were also investigated; however, these results were not remarkable compared to those of the conductivity. Thus, the results of this study present a significant correction formula for temperature when noninvasively measuring blood glucose levels using electrical impedance spectroscopy. Because this value is considered to be independent of frequency within the tested frequency range (1 kHz to 110 MHz), it can also be used universally for temperature corrections for measuring blood impedance. In the future, we intend to conduct experiments that subdivide the blood glucose levels range from hypoglycemia to hyperglycemia because hypoglycemia is an issue as important as hyperglycemia in managing diabetes. Furthermore, significant results are expected to be obtained when the proposed method is applied to mixtures of other bio-samples.

## Supplementary Materials

The following are available online at https://www.mdpi.com/1424-8220/20/21/6231/s1: Figure S1: Glucose dependence of conductivity in plasma and cytoplasm, Figure S2: Temperature dependence of permittivity in cytoplasm and membrane, Figure S3: Trends of permittivity in cytoplasm and membrane according to glucose level, Table S1: Temperature dependence of conductivity in plasma and cytoplasm for each subject.
