# Supplementary material for: Temperature Correction to Enhance Blood Glucose Monitoring Accuracy Using Electrical Impedance Spectroscopy
Source: Sensors (Basel). 2020 Oct 31;20(21):6231. doi: 10.3390/s20216231 (PMC7663582; doi:10.3390/s20216231)
Supplement: Supplementary file 1 [file sensors-20-06231-s001.pdf]

Temperature Correction to Enhance Blood Glucose Monitoring Accuracy Using Electrical Impedance Spectroscopy

Ye Sung Lee [1], Minkook Son [2], Alexander Zhbanov [1], Yugyung Jung [2], Myoung Hoon Jung [3], Kunsun Eom [3], Sung Hyun Nam [3], Jongae Park [3], and Sung Yang [1,2]*

[1] School of Mechanical Engineering, Gwangju Institute of Science and Technology (GIST), Gwangju 61005, Republic of Korea
[2] Department of Biomedical Science and Engineering, Gwangju Institute of Science and Technology (GIST), Gwangju 61005, Republic of Korea
[3] Healthcare Sensor Lab, Device Research Center, Samsung Advanced Institute of Technology, Samsung Electronics Co. Ltd., 130 Samsung-ro, Yeongtong-gu, Suwon-si, Gyeonggi-do 16678, Republic of Korea
* Correspondence: syang@gist.ac.kr; Tel.: +82-62-715-2407 (S.Y.); Fax: +82-62-715-2384

Received: date; Accepted: date; Published: date

Supplementary materials

Figure S1. Glucose dependence of conductivity in plasma and cytoplasm.
Figure S2. Temperature dependence of permittivity in cytoplasm and membrane.
Figure S3. Trends of permittivity in cytoplasm and membrane according to the glucose level.
Table S1. Temperature dependence of conductivity in plasma and cytoplasm for each subject.

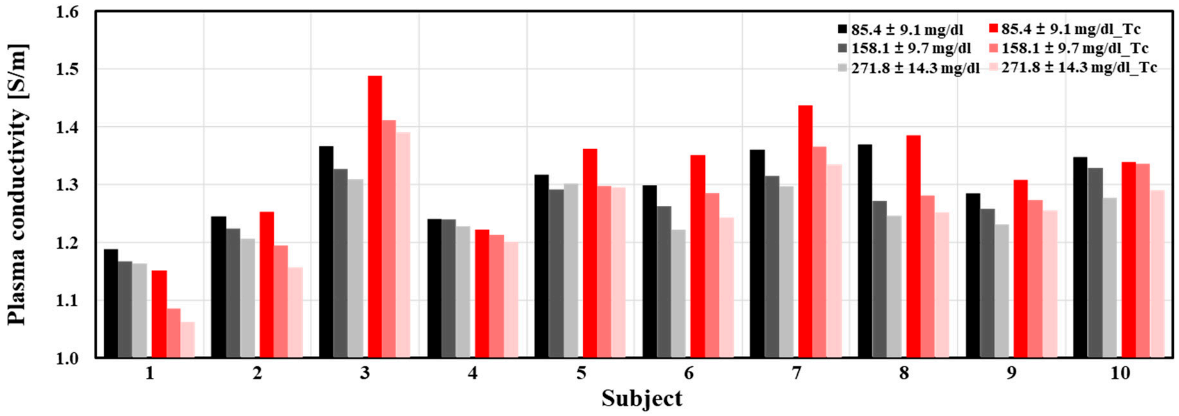

(**a**)

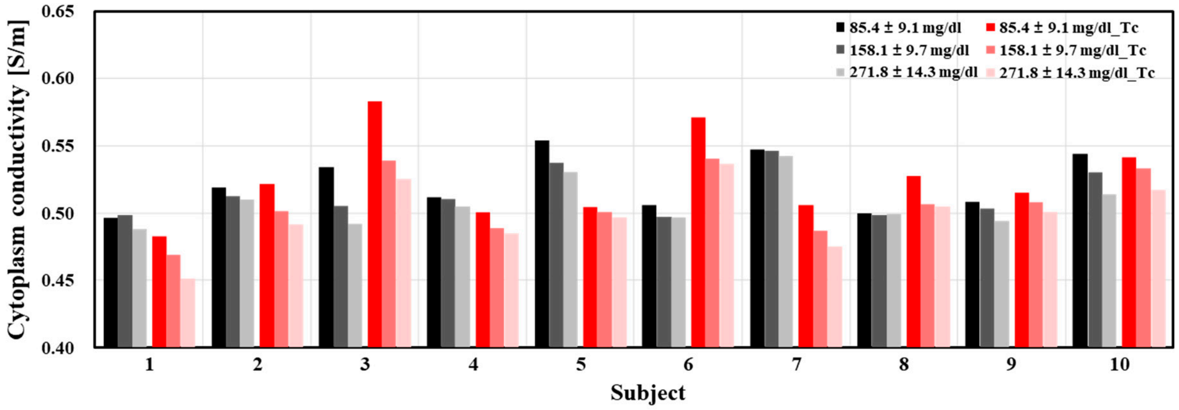

(**b**)

Figure S1. Glucose dependence of conductivity in plasma and cytoplasm. From 10 subjects, the electrical conductivities of plasma and cytoplasm are extracted with three glucose levels: 85.4±9.1mg/dl, 158.1±9.7mg/dl, and 271.8±14.3mg/dl. (**a**) Plasma conductivity. The electrical conductivity of plasma tends to decrease as glucose level increases. Before temperature correction data are gray blocks, and after temperature correction data are red blocks. (**b**) Cytoplasm conductivity. The electrical conductivity of the cytoplasm tends to decrease as the glucose level increases. Before temperature correction data are gray blocks, and after temperature correction data are red blocks.

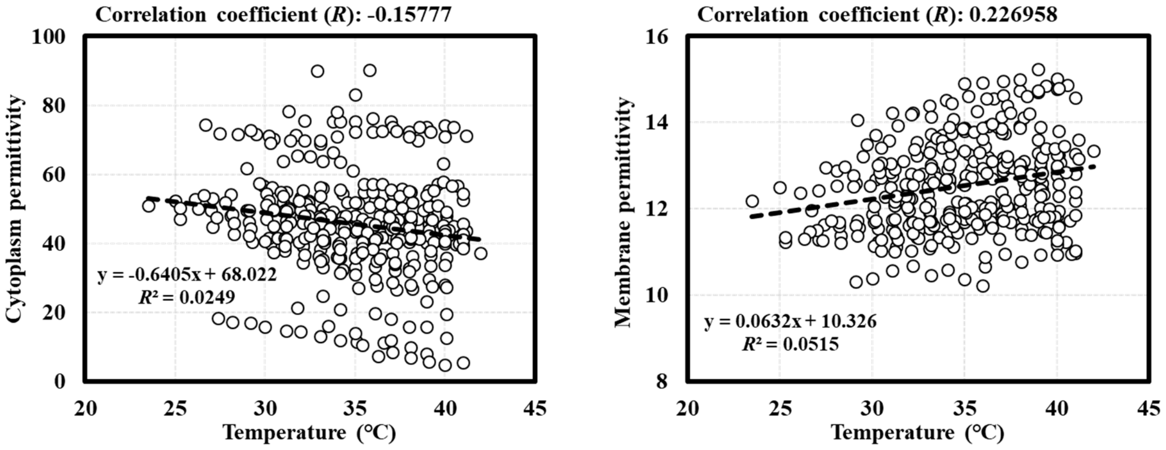

(a) (b)

Figure S2. Temperature dependence of permittivity in cytoplasm and membrane. (**a**) Cytoplasm permittivity. Correlation analysis and linear regression analysis are performed. The correlation coefficient(R) is -0.158, and it means that it is difficult to specify the tendency with temperature. The R^2 value of the linear regression analysis is 0.02. (**b**) Membrane permittivity. Correlation analysis and linear regression analysis are performed. The correlation coefficient (R) is 0.227, and it means that it is difficult to specify the tendency with temperature. The R^2 value of the linear regression analysis is 0.05.

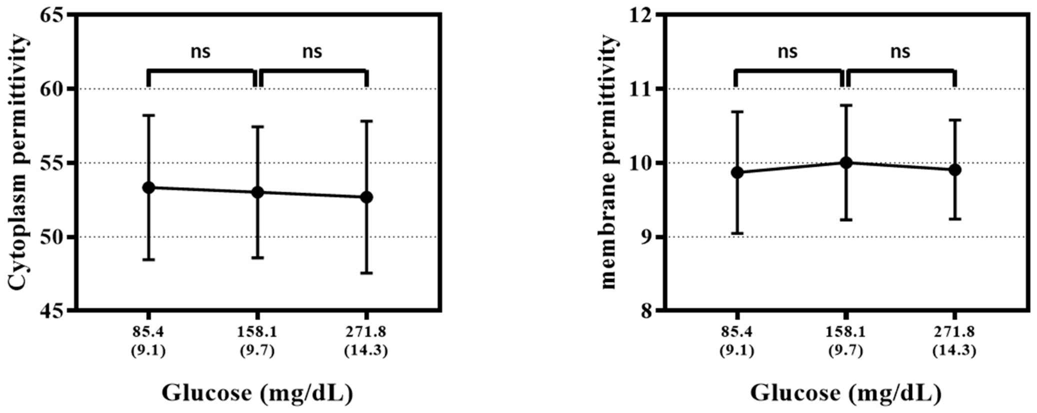

(a) (b)

Figure S3. Trends of permittivity in cytoplasm and membrane according to glucose level. The permittivity of cytoplasm and membrane has no significant relation with glucose level (ns: not significant). (**a**) Cytoplasm permittivity with increasing glucose level. (**b**) Membrane permittivity with increasing glucose level.

Table S1. Temperature dependence of conductivity in plasma and cytoplasm for each subject.

	Plasma conductivity [S/m]		Cytoplasm conductivity [S/m]	
**Subject**	R^2	**Equation**	R^2	**Equation**
201901	0.9402	y = 0.0382x + 0.281	0.8805	y = 0.0141x + 0.2094
201902	0.9512	y = 0.0396x + 0.2909	0.9168	y = 0.0154x + 0.1572
201903	0.8415	y = 0.0402x + 0.1362	0.6966	y = 0.0163x + 0.0888
201904	0.9419	y = 0.0326x + 0.4454	0.8836	y = 0.013x + 0.2133
201905	0.9561	y = 0.0374x + 0.3551	0.8762	y = 0.0144x + 0.1932
201906	0.9555	y = 0.0418x + 0.2957	0.6698	y = 0.0133x + 0.2423
201907	0.9262	y = 0.0369x + 0.3837	0.6556	y = 0.0138x + 0.2068
201908	0.9458	y = 0.0443x + 0.0847	0.9248	y = 0.018x + 0.0829
201909	0.806	y = 0.0384x + 0.2668	0.7443	y = 0.017x + 0.1461
201910	0.94	y = 0.0447x + 0.1949	0.8926	y = 0.013x + 0.2837
